# Development and performance assessment of novel machine learning models to predict pneumonia after liver transplantation

**DOI:** 10.1186/s12931-021-01690-3

**Published:** 2021-03-31

**Authors:** Chaojin Chen, Dong Yang, Shilong Gao, Yihan Zhang, Liubing Chen, Bohan Wang, Zihan Mo, Yang Yang, Ziqing Hei, Shaoli Zhou

**Affiliations:** 1 Department of Anesthesiology, The Third Affiliated Hospital of Sun Yat-Sen University, No. 600 Tianhe Road, Guangzhou, 10630 Guangdong People’s Republic of China; 2 Guangzhou AID Cloud Technology Co., LTD, Guangzhou, People’s Republic of China; 3 Department of Information, The Third Affiliated Hospital of Sun Yat-Sen University, Guangzhou, People’s Republic of China; 4 Department of Hepatic Surgery and Liver Transplantation Center, The Third Affiliated Hospital of Sun Yat-Sen University, No. 600 Tianhe Road, Guangzhou, 510630 Guangdong People’s Republic of China

**Keywords:** Liver transplantation, Postoperative pneumonia, Machine learning, Postoperative pulmonary complications, Disease prediction, Risk factors, Early intervention, Deep learning, ML algorithm, Extreme gradient boosting

## Abstract

**Background:**

Pneumonia is the most frequently encountered postoperative pulmonary complications (PPC) after orthotopic liver transplantation (OLT), which cause high morbidity and mortality rates. We aimed to develop a model to predict postoperative pneumonia in OLT patients using machine learning (ML) methods.

**Methods:**

Data of 786 adult patients underwent OLT at the Third Affiliated Hospital of Sun Yat-sen University from January 2015 to September 2019 was retrospectively extracted from electronic medical records and randomly subdivided into a training set and a testing set. With the training set, six ML models including logistic regression (LR), support vector machine (SVM), random forest (RF), adaptive boosting (AdaBoost), extreme gradient boosting (XGBoost) and gradient boosting machine (GBM) were developed. These models were assessed by the area under curve (AUC) of receiver operating characteristic on the testing set. The related risk factors and outcomes of pneumonia were also probed based on the chosen model.

**Results:**

591 OLT patients were eventually included and 253 (42.81%) were diagnosed with postoperative pneumonia, which was associated with increased postoperative hospitalization and mortality (*P* < 0.05). Among the six ML models, XGBoost model performed best. The AUC of XGBoost model on the testing set was 0.734 (sensitivity: 52.6%; specificity: 77.5%). Pneumonia was notably associated with 14 items features: INR, HCT, PLT, ALB, ALT, FIB, WBC, PT, serum Na^+^, TBIL, anesthesia time, preoperative length of stay, total fluid transfusion and operation time.

**Conclusion:**

Our study firstly demonstrated that the XGBoost model with 14 common variables might predict postoperative pneumonia in OLT patients.

**Supplementary Information:**

The online version contains supplementary material available at 10.1186/s12931-021-01690-3.

## Introduction

Postoperative pulmonary complications (PPC) adversely affect the clinical course of orthotopic liver transplantation (OLT) and play an important role in poor survival [1]. Postoperative pneumonia is the most common type of PPC, contributing to morbidity, length of hospital stay, and mortality [2]. Identification of patients at high risk of developing postoperative pneumonia is the key to early implementing interventions to prevent its onset or antibiotics to treat bacterial infection [3]. On the contrary, unnecessary and excessive antibiotic use in patients at low risk for postoperative pneumonia can lead to antibiotic resistance and side effects. For instance, recent studies have shown that extensive use of antibiotics for anti-bacteria prophylaxis, multi-drug resistant bacteria in post-transplant patients have been induced [4, 5]. Therefore, it is essential to establish a reliable model for prediction of postoperative pneumonia to tailor preventive interventions and treatments for patients at high-risk of postoperative pneumonia and avoid unnecessary use of antibiotics in low-risk patients.

In recent years, several scoring systems for prediction of postoperative pneumonia have been reported to improve risk-stratification [6], such as the Prestroke Independence, Sex, Age, National Institutes of Health Stroke Scales (ISAN) in acute ischemic stroke patients [7], a pneumonia risk index for patients undergoing major noncardiac surgery [8], and a systemic inflammation score for patients after radical resection of gastric cancer [9, 10]. However, these predictive models are not applicable to liver transplant recipients, mainly due to the preoperative pulmonary condition of patients with end-stage liver disease and the immunosuppressive status of allograft recipients [10]. Currently, an effective risk classification for postoperative pneumonia has not yet been available for liver transplant recipients.

Compared with the traditional scoring systems, machine learning (ML) models have shown better performance in predicting various diseases or clinical conditions [11–13]. ML models are usually constructed based on high volume data recorded in the electronic patient record (EPR) systems and its deep learning ability allows ML models to capture complex, nonlinear relationships, even previously unknown correlations in big data, digging deeper into clinical data [14], and shows promising potential in clinical scenes where large amount of data were collected and integrated every day. Recently, Li and colleagues [15] have developed a model using ML methods to predict stroke-associated pneumonia in Chinese patients with acute ischemic stroke. In addition, ML was used to predict severe pneumonia during posttransplant hospitalization in recipients of a kidney transplant [16]. ML was also applied in developing models for liver disease and transplantation to predict post-transplant survival and complications, including acute kidney injury (AKI) and diabetes [17]. To date, there has been no ML model for prediction of postoperative pneumonia in recipients of liver transplant [18].

In this study, we aimed to develop predictive models using ML methods, and to evaluate their performance in predicting postoperative pneumonia in OLT patients. The findings obtained through conducting this study was expected to provide a novel ML algorithm for prediction of postoperative pneumonia in patients after liver transplantation.

## Materials and methods

### Human subjects and study design

In this retrospective study, data of 894 patients who underwent either living donor liver transplantation (LDLT) or deceased donor liver transplantation (DDLT) in the Third Affiliated Hospital of Sun Yat-sen University-Lingnan Hospital (Guangzhou, Guangdong, China) spanning from January 2015 to September 2019 were retrieved from the EPR systems. All the patients were registered as recipients of organ transplantation in the China Organ Transplant Response Systems (www.cot.org.cn). During the retrospective enrollment, the patients aged < 18 years, presented with preoperative pneumonia or lack of sufficient post-operative data were excluded from this study.

In the EPR systems of our hospital, a database platform was established by extracting medical records from hospital information system (HIS), laboratory information system (LIS), picture archiving and communication system (PACS), and Docare Anesthesia System (2005–2020 Medicalsystem Co., Ltd. Suzhou, China). This database platform enabled access to comprehensive data collected during hospital admission, inpatient stay, and post-hospital follow-up visit, including demographic characteristics, daily documentation, laboratory tests, imaging results, anesthesia records, and other clinical characteristics. This study was reported in accordance with the Transparent Reporting of a Multivariable Prediction Model for Individual Prognosis or Diagnosis (TRIPOD) guidelines.

### Primary outcome

The primary outcome was the incidence of postoperative pneumonia during the postoperative period before hospital discharge. Postoperative pneumonia was defined on the basis of European Perioperative Clinical Outcome (EPCO) definitions, in which at least one of the following definitive chest X-ray or CT findings was fulfilled: infiltrate, consolidation, cavitation; and at least one of the following signs and symptoms of infection (Temperature > 38 °C or < 36 °C with no other causes, white blood cell (WBC) count > 10 × 10^9^/L or < 4 × 10^9^/L)[6].

### Data selection

The data elements related to the following categories were chosen from database platform: (1) Demographics: age, gender, height and weight; (2) Preoperative comorbidities: hypertension, coronary heart disease, myocardial infarction, diabetes mellitus, history of alcohol abuse, smoking, and past surgery; (3) Etiology: primary liver diseases contributing to the decision of LT with main focus on hepatitis B, hepatitis C, dual infection of any combination of the known hepatitis virus A to E, hepatic malignancy (including hepatocellular carcinoma and cholangiocarcinoma), alcohol-related liver disease (ALD), drug-induced liver injury (DILI), and autoimmune liver disease; (4) Perioperative laboratory values: lab results concerning liver function, kidney function, electrolytes, and count of blood cells. The results of the latest tests prior to surgery were collected. Lab MELD score prior to surgery was calculated; (5) Preoperative complications: complications and metrics reminding the severity of the patients were collected, which mainly consist of complications related to cirrhosis and portal hypertension, the documentation of treatment escalation including length of stay in ICU, use of continuous blood purification (CBP) and mechanical ventilation; (6) Intraoperative incidents: incidents indicating hemodynamic instability, such as cardia arrest, arrhythmia, lactic acidosis, acidosis, hypernatremia, hypokalemia, and hypotension; (7) Intraoperative medication: including intraoperative use of vasoconstrictors (either used as bolus or continuously) and blood coagulant, which reflected the extent of hemodynamic instability and hemorrhagic tendency. The data collected were the accumulative sum by the end of the surgery; (8) Intraoperative fluid and transfusion: the total of intraoperative fluid infusion and output, as well as the total of blood product transfused were respectively extracted. Red blood cell transfusion, plasma transfusion, total blood product transfusion and total fluid transfusion were all classified into two categories based on specific criterions; (9) Post-operative medications with mainly traced the post-operative medications within 7 days after surgery. These medications consist of colloid, vasoconstrictors, as well as immunosuppressant, antifungal agents and antibiotics; (10) Microorganism observation: test on microorganism during preoperative period and post-operative period.

### Variable selection

With 591 records and 148 features, overfitting could occur during training and undermine model performance. Therefore, we first implemented univariate test to filter out features that were statistically insignificant. Finally, 33 features were statistically significant (*P* < 0.05) and proceeded to be used in a recursive feature elimination (RFE) method embedded with random forest [19]. Initially, RFE method trained on all features and then it recursively removed least important features, the subset of features which had the highest sensitivity score was selected.

### Development of machine learning models

To predict postoperative pneumonia, the following six different machine learning models were developed and evaluated for their performance: logistic regression (LR) [20], support vector machine (SVM) [21], random forest (RF) [22], MLP (multilayer perceptron) [23], extreme gradient boosting (XGBoost) [24], and gradient boosting machine (GBM) [25].

XGBoost model was constructed using the xgboost package (https://xgboost.readthedocs.io/en/latest/python/index.html). The remaining five models were established via Scikit-learn package (https://github.com/scikit-learn/scikit-learn). Considering that machine learning models had multiple tuning parameters which were essential for model performance, fivefold cross-validation grid search method was used for selection of the best parameters and AUCs on testing set were measured (Additional file 1: Table S1). The complete data set of 591 adult was then randomly separated into 70% train and 30% test for validation. Bootstrap method was then used to sample 1000 different test sets in order to get 95% confidence interval (CI) of the best tuned models’ evaluation metrics. Model performance was evaluated by area under receiver-operating curve (AUC), accuracy, sensitivity, and specificity.

### Statistical analysis

Python (Anaconda Distribution, version 3.7) package Numpy (version 1.16.5) and Pandas (version 0.25.1) were employed for data cleaning. Python (Anaconda Distribution version 3.7) Scipy package (version 1.3.1) were used to analyze the data. The continuous variables were presented with the mean along with standard deviation (SD), or median along with interquartile range. Independent sample t-test was used for normally distributed data, while Mann–Whitney U test was used for non-normal distribution data in univariate analyses. Categorical variables were expressed with quantities and percentages, and tested by Chi-square test or Fisher’s exact test. Kaplan–Meier methods were applied to estimate the long-term survival rates. Besides, the comparisons between groups were performed by Gehan–Breslow–Wilcoxon test and Log-rank test.

No variables had missing percentage higher than 1%. We employed mean imputation, which imputed missing value with the mean of each feature, to fill in missing values. Before we proceeded to machine learning models, continuous variables were normalized based on the mean and SD of the training set. Categorical variables were encoded into binary variable, 1 represents having an incident, 0 represents not having an incident. Gender was also encoded, 1 represents male, 0 represents female. The whole dataset was split into 70% of training set and 30% of testing set. The data in the training set was used for development of predictive models, while the testing set was used to validate models’ performance.

## Results

### Characteristics of the study subjects and preoperative factors associated with postoperative pneumonia

**Fig. 1:**
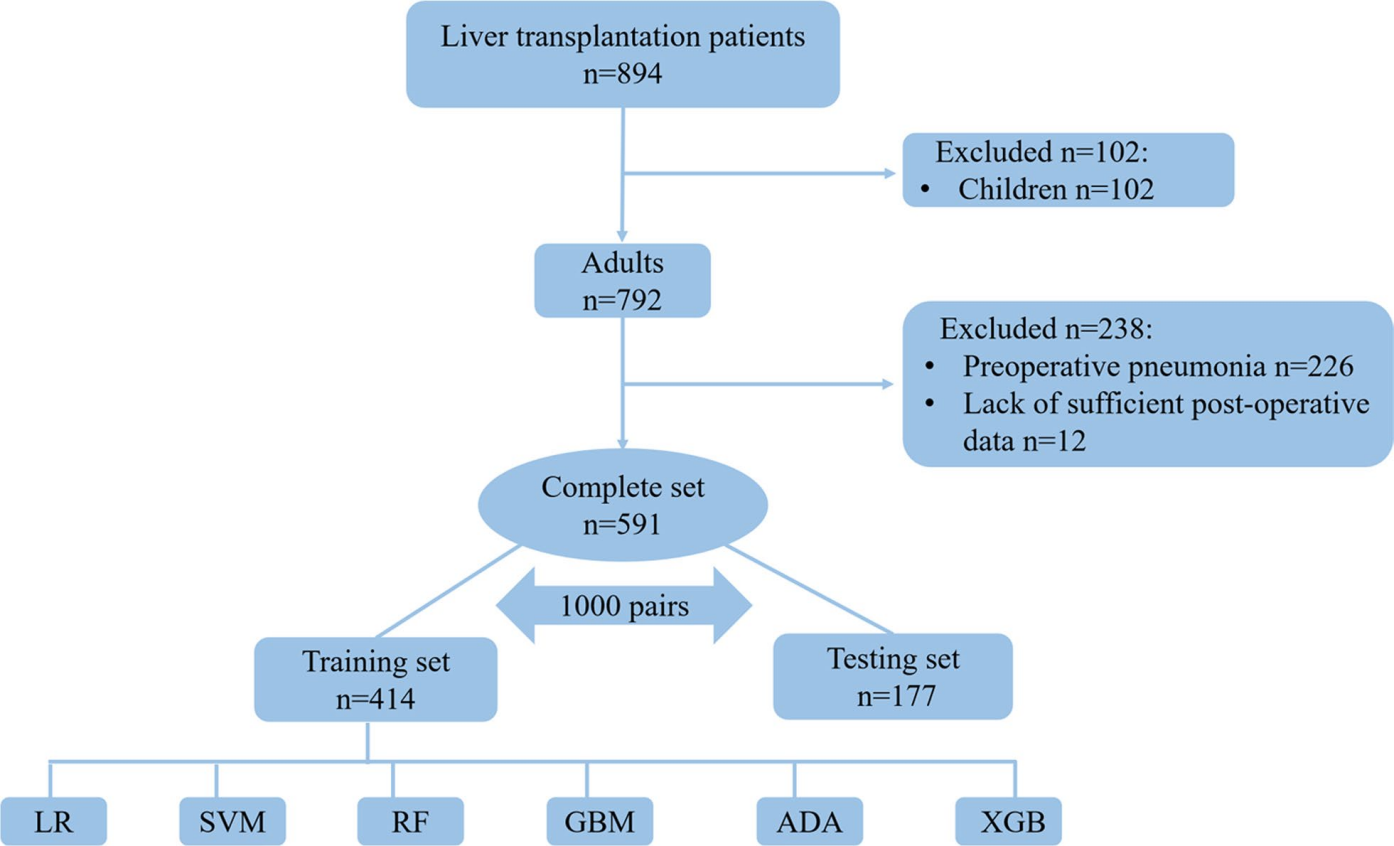
Flow chart of patient enrollment in this study. *LR* logistic regression, *SVM* support vector machine, *RF* random forest, *GBM* gradient boosting machine, *MLP* multilayer perceptron, *XGB* extreme gradient boosting

A total of 894 patients who underwent orthotopic liver transplantation in our hospital, spanning the period from January 2015 to September 2019, were assessed for eligibility. After 65 pediatric patients, 226 patients with preoperative pneumonia, and 12 patients lack of sufficient postoperative data, were excluded, 591 patients were finally enrolled and used for development and performance evaluation of machine learning models to predict postoperative pneumonia. The flow diagram of the enrollment was presented in Fig. 1. Notably, pneumonia occurred in 253 patients, accounting for as high as 42.81% of the study subjects following liver transplantation, while 338 (57.19%) patients did not have postoperative pneumonia.

The demographic characteristics, laboratory tests results, and clinical features of the enrolled patients with or without postoperative pneumonia were summarized in Table 1. The demographic characteristics and preoperative comorbidities did not differ significantly between the patients with or without occurrence of postoperative pneumonia (*P* > 0.05). Notably, hepatic malignancy, hematocrit (HCT), alanine transaminase (ALT), total bilirubin (TBIL), albumin (ALB), coagulation function, MELD score, and hospital stay were found to have significant differences between patients with or without postoperative pneumonia (*P* < 0.05). In particular, the patients without postoperative pneumonia had significantly better preoperative hepatic function, as reflected by preoperative liver function tests in comparison with those patients who developed pneumonia after surgery (*P* < 0.05).

**Table 1:** Preoperative characteristics of the study patients

Variables	Patients without pneumonia (n = 338)	Patients with pneumonia (n = 253)	*P*_value
Gender			0.377
Male	303 (0.896)	220 (0.87)	
Female	35 (0.104)	33 (0.13)	
Height (cm)	168.45 (6.285)	167.812 (13.067)	0.997
Weight (kg)	64.803 (11.661)	65.24 (10.416)	0.373
Body Mass Index	22.836 (3.676)	22.855 (3.521)	0.527
Age (y)	50.68 (11.129)	50.423 (10.794)	0.778
Comorbidities			
Hypertension (n)	30 (0.089)	28 (0.111)	0.455
Diabetes mellitus (n)	54 (0.16)	30 (0.119)	0.194
Myocardial infarction (n)	0 (0.0)	0 (0.0)	1
Coronary artery disease (n)	5 (0.015)	1 (0.004)	0.376
History of smoking (n)	86 (0.254)	72 (0.285)	0.468
Alcohol abuse (n)	88 (0.26)	51 (0.202)	0.117
Previous surgical history (n)	20 (0.059)	24 (0.095)	0.14
Etiology for liver transplantation			
Hepatitis B (n)	252 (0.746)	196 (0.775)	0.471
Hepatitis C (n)	8 (0.024)	3 (0.012)	0.457
Dual infection (n)	4 (0.012)	2 (0.008)	0.955
Hepatic malignancy (n)	129 (0.382)	123 (0.486)	**0.014**
Drug-induced liver injury (n)	5 (0.015)	2 (0.008)	0.703
Alcohol-related liver disease (n)	16 (0.047)	5 (0.02)	0.117
Auto-immune hepatitis (n)	1 (0.003)	2 (0.008)	0.801
Hepatolenticular degeneration (n)	2 (0.006)	4 (0.016)	0.44
Hemochromatosis (n)	0 (0.0)	0 (0.0)	1
Laboratory results			
Hematocrit (HCT)	0.323 (0.082)	0.299 (0.071)	**< 0.001**
Platelets (10^9^/L)	99.935 (85.816)	104.597 (75.634)	0.105
WBC (10^9^/L)	6.844 (5.013)	7.344 (4.183)	**0.002**
WBC > 11.2 * 10^9^/L (n)	45 (0.133)	33 (0.13)	0.979
ALT (U/L)	99.251 (201.942)	192.17 (609.322)	**0.046**
AST (U/L)	124.861 (223.034)	268.905 (850.699)	0.625
TBIL (μmol/L)	224.753 (262.801)	263.291 (256.749)	**0.008**
IBIL (μmol/L)	92.198 (96.445)	88.781 (106.336)	0.071
ALB (g/L)	36.71 (5.009)	35.73 (4.953)	**0.008**
Last SCr (μmol/L)	89.571 (71.798)	92.911 (76.955)	0.675
BUN (mmol/L)	6.539 (5.074)	6.214 (5.313)	0.356
PT (s)	23.411 (13.079)	25.313 (12.233)	**0.005**
APTT (s)	51.496 (20.168)	54.365 (18.819)	**0.006**
FIB (g/L)	1.904 (1.226)	2.324 (1.792)	**0.001**
INR	2.163 (1.651)	2.396 (1.505)	**0.003**
Serum potassium (mmol/L)	3.865 (0.509)	3.829 (0.497)	0.396
Serum sodium (mmol/L)	138.338 (5.065)	139.489 (5.409)	**0.015**
Serum calcium (mmol/L)	2.328 (0.217)	2.35 (0.209)	0.106
HCO3− (mmol/L)	22.698 (3.563)	23.21 (6.124)	0.551
Complications and treatments			
MELD score	16 (9–29)	30 (17–38)	**< 0.001**
Cirrhosis (n)	273 (0.808)	190 (0.751)	0.12
Primary biliary cirrhosis (n)	3 (0.009)	1 (0.004)	0.83
Alcoholic liver cirrhosis (n)	10 (0.03)	3 (0.012)	0.242
Hepato-renal syndrome (n)	12 (0.036)	9 (0.036)	0.826
Hepatopulmonary syndrome (n)	0 (0.0)	0 (0.0)	1
Hepatic encephalopathy (n)	69 (0.204)	59 (0.233)	0.455
Portal hypertension (n)	180 (0.533)	119 (0.47)	0.158
Ascites (n)	137 (0.405)	95 (0.375)	0.516
Preoperative length of stay (d)	12 (4–27)	3 (0–15)	**< 0.001**
Preoperative ICU stay (n)	185 (0.547)	147 (0.581)	0.464
Preoperative dialysis (n)	0 (0.0)	2 (0.008)	0.357
Preoperative continuous blood purification	51 (0.151)	32 (0.126)	0.468
Mechanical ventilation (n)	19 (0.056)	10 (0.04)	0.461
Hypokalemia (n)	81 (0.24)	59 (0.233)	0.933
Hyperkalemia (n)	0 (0.0)	0 (0.0)	1
Hyponatremia (n)	81 (0.24)	36 (0.142)	**0.005**
Hypernatremia (n)	15 (0.044)	24 (0.095)	**0.023**
Hypocalcemia (n)	0 (0.0)	0 (0.0)	1
Hypercalcemia (n)	13 (0.038)	11 (0.043)	0.924
Metabolic acidosis (n)	149 (0.441)	103 (0.407)	0.462

Data were expressed as frequency (proportion). Continuous variables were presented as mean (standard deviation), or median (interquartile range).  The bold emphasis means that *p* < 0.05

*WBC* white blood cell, *ALT* alanine transaminase, *AST* aspartate amino transferase, *TBIL* total bilirubin, *IBIL* indirect bilirubin, *ALB* albumin, *BUN* blood urea nitrogen, *PT* prothrombin time, *APTT* activated partial thromboplastin time, *FIB* fibrinogen, *INR* international normalized ratio

### Analysis of intraoperative and postoperative factors related to postoperative pneumonia

The intraoperative factors, including those in the following three categories: intraoperative incidents, fluid management and transfusion, and medications, were compared between the study patients with or without postoperative pneumonia. As shown in Table 2, hypernatronemia, longer operation time and anesthesia time, more red blood cell (RBC) transfusion and blood product transfusion, larger volume of infusion and more blood loss were found to be significantly associated with postoperative pneumonia (*P* < 0.05). Notably, the proportions of patients with RBC transfusion > 18U, blood product transfusion > 5000 mL, total volume of infusion > 10 L, and blood loss > 2 L were significantly higher in the pneumonia group than the non-pneumonia group (Table 2). In addition, higher doses of recombinant activated factor VII (0.343 ± 1.031 vs. 0.134 ± 0.615, *P* = 0.008) and prothrombin complex concentrate (602.367 ± 410.826 vs. 506.719 ± 359.224, *P* = 0.01) were administrated in the patients without pneumonia than those with pneumonia.

**Table 2:** Comparison of intraoperative factors between patients with or without postoperative pneumonia

Variables	Patients without pneumonia (338)	Patients with pneumonia (253)	*P*_value
Intraoperative incidents			
Arrhythmia (n)	330 (0.976)	247 (0.976)	0.787
Cardiac arrest (n)	12 (0.036)	3 (0.012)	0.123
Acidosis (n)	139 (0.411)	104 (0.411)	0.936
Hyperlactacidemia (n)	166 (0.491)	133 (0.526)	0.454
Hypokalemia (n)	135 (0.399)	106 (0.419)	0.693
Hypernatronemia (n)	4 (0.012)	11 (0.043)	**0.031**
Hypotension (n)	276 (0.817)	215 (0.85)	0.339
Warm ischemic time (min)	45.317 (11.688)	47.36 (12.055)	0.095
Cold ischemic time (h)	6.269 (1.383)	6.289 (1.41)	0.838
Operation time (min)	434.723 (118.926)	452.512 (126.181)	**0.044**
Anesthesia time (min)	527.146 (119.563)	549.837 (132.364)	**0.011**
Intraoperative fluid management and transfusion			
Crystalloid (mL)	2412.151 (1939.289)	2632.789 (2193.884)	0.305
Colloid (mL)	94.622 (221.09)	126.855 (528.29)	0.619
RBC transfusion (mL)	1165.386 (999.811)	1510.142 (1199.629)	**< 0.001**
RBC transfusion > 18U	28 (0.083)	64 (0.253)	**< 0.001**
Plasma transfusion (mL)	1674.701 (1512.438)	1834.57 (1545.028)	0.212
Plasma transfusion > 3000 mL	36 (0.142)	61 (0.18)	0.259
Cryoprecipitate transfusion (U)	28.772 (15.457)	29.634 (14.761)	0.572
Cryoprecipitate > 35U	90 (0.356)	113 (0.334)	0.649
Sodium bicarbonate transfusion (mL)	94.209 (188.062)	131.598 (252.291)	0.109
Albumin (mL)	218.048 (111.48)	226.988 (123.224)	0.387
Other fluids (mL)	74.815 (343.199)	41.006 (206.839)	0.431
Blood product transfusion (mL)	3252.028 (2035.43)	3768.967 (2161.842)	**0.002**
Blood product transfusion > 5000 mL	35 (0.104)	77 (0.304)	**< 0.001**
Total volume of infusion (mL)	6115.221 (3632.741)	6926.488 (4323.961)	**0.024**
Total volume of infusion > 10 L	24 (0.071)	55 (0.217)	**< 0.001**
Blood loss (mL)	1740.913 (1767.973)	2031.361 (1768.054)	**0.006**
Blood loss > 2 L	59 (0.175)	106 (0.419)	**< 0.001**
Urine output (mL/(kg h))	3.367 (2.311)	3.165 (2.058)	0.457
Ascites removal (mL)	817.769 (1836.427)	939.318 (1825.486)	0.073
Gastric drainage (mL)	71.773 (278.9)	51.157 (119.586)	0.988
Other estimated fluid loss (mL)	6.883 (77.52)	3.697 (33.576)	0.215
Intraoperative medications			
Recombinant activated factor VII (mg)	0.343 (1.031)	0.134 (0.615)	**0.008**
Prothrombin complex concentrate (IU)	602.367 (410.826)	506.719 (359.224)	**0.01**
Use of dopamine, continuous (n)	102 (0.302)	80 (0.316)	0.775
Use of metaraminol, continuous (n)	6 (0.018)	3 (0.012)	0.811
Use of norepinephrine, continuous (n)	281 (0.831)	216 (0.854)	0.533
Use of epinephrine, continuous (n)	246 (0.728)	176 (0.696)	0.445

Data were expressed as frequency (proportion) or median (IQR). Continuous variables were presented with mean along with standard deviation (SD), or median (interquartile range). The bold emphasis means that *p* < 0.05

*IV* intravenous injection

In terms of postoperative medications (Table 3), the doses of telipressin and dopamine in patients without pneumonia were significantly higher than those with pneumonia (0.148 ± 0.414 vs. 0.079 ± 0.314 mg/day, *P* = 0.012; 47.544 ± 72.198 vs. 35.473 ± 63.069 mg/day, *P* = 0.013; respectively). There were no significant differences between the two groups in terms of norepinephrine, dopamine, epinephrine and tacrolimus (*P* > 0.05).

**Table 3:** Comparison of postoperative features of the study patients

Variables	Patients without pneumonia (338)	Patients with pneumonia (253)	*P*_value
Dose of norepinephrine (mg/day)	5.079 (10.559)	3.431 (8.144)	**0.055**
Use of norepinephrine, continuous (n)	60 (0.178)	33 (0.13)	0.15
Dose of telipressin (mg/day)	0.148 (0.414)	0.079 (0.314)	**0.012**
Use of dopamine, continuous (n)	85 (0.251)	46 (0.182)	0.055
Dose of dopamine (mg/day)	47.544 (72.198)	35.473 (63.069)	**0.013**
Use of epinephrine, continuous (n)	7 (0.021)	2 (0.008)	0.358
Dose of epinephrine (mg/day)	1.883 (3.91)	1.469 (3.011)	0.143
Tacrolimus (n)	3 (0.009)	1 (0.004)	0.83
Postoperative hospitalization (day)	22 (17, 31)	23 (17, 33)	**0.046**
Total hospitalization (day)	39 (24, 53)	32 (20, 48)	**0.008**
Total cost (yuan)	301,467 (244,611, 394,379)	294,620 (244,519, 377,520)	0.418

Data were expressed as frequency (proportion) or median (IQR). Continuous variables were presented with mean along with standard deviation (SD), or median (interquartile range). The bold emphasis means that *p* < 0.05

### Feature selection using univariate and recursive feature elimination methods

**Fig. 2:**
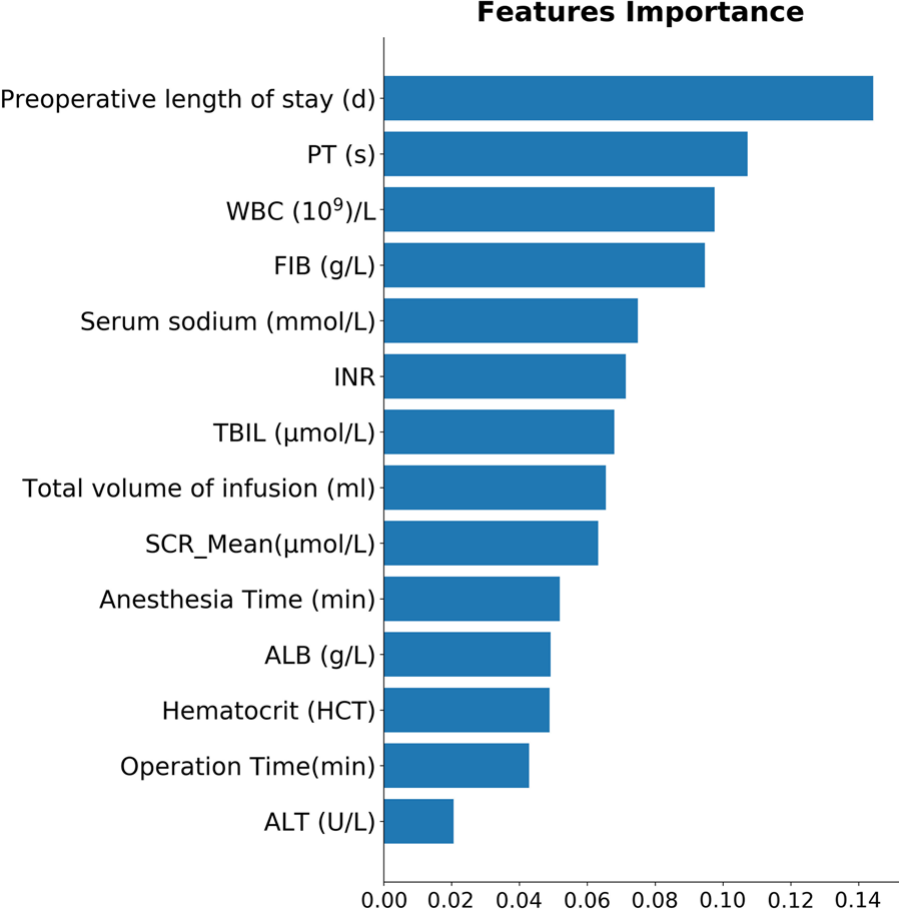
Feature importance ranking of the selected 14 features illustrated by random forest. *PT* prothrombin time, *WBC* white blood cells, *FIB* fibrinogen, *INR* international normalized ratio, *TBIL* total bilirubin, *SCR* serum creatinine, *ALB* albumin, *HCT* hematocrit, *ALT* glutamic pyruvic transaminase

As partially relevant or less important features may negative affect performance of machine learning models, we performed feature selection and ranked levels of feature importance. Feature selection was performed using univariate and recursive feature elimination (RFE) methods, after which dimensionality was reduced from 148 to 14 features. These 14 features were listed as follows: preoperative international normalized ratio (INR), HCT, platelets (PLT), ALB, ALT, fibrinogen (FIB), WBC, prothrombin time (PT), serum sodium (Na^+^), TBIL, anesthesia time, preoperative hospital stay, total fluid transfusion, and operation time. Further, feature importance plot was created to rank the levels of importance using fine tuned eXtreme Gradient Boosting (XGBoost) model. As a result, preoperative length of hospital stay, PT, and WBC were ranked first, second, and third, respectively (Fig. 2).

### Performance assessment of the machine learning models for prediction of postoperative pneumonia

**Fig. 3:**
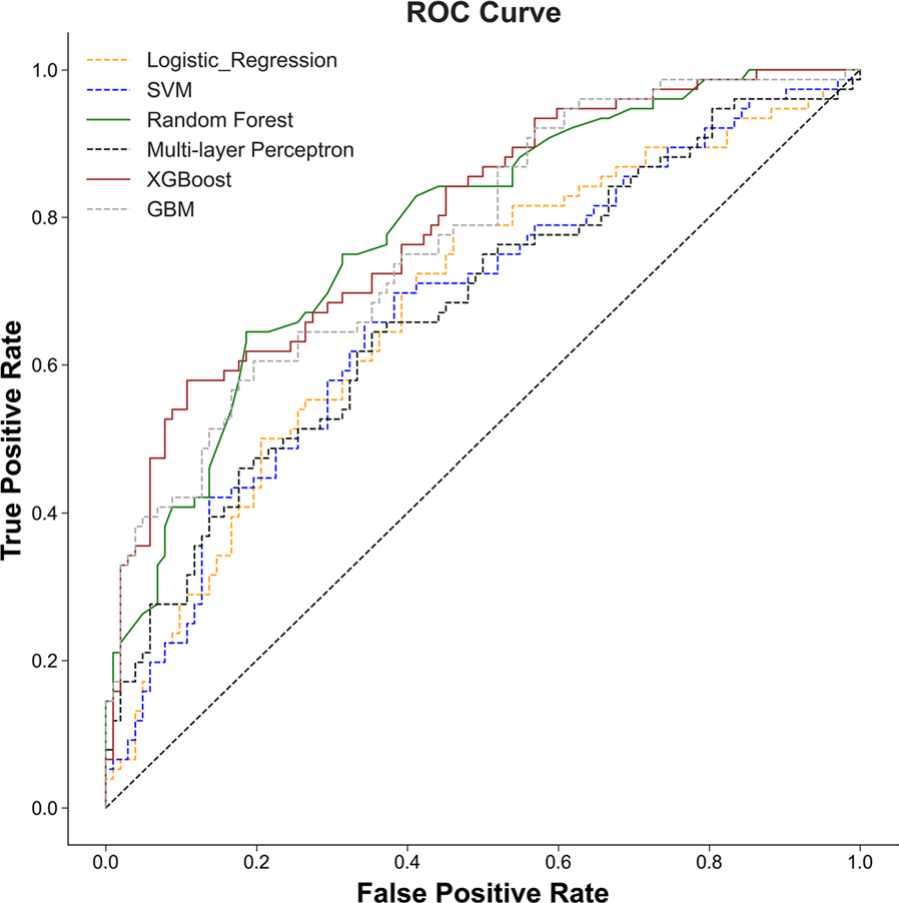
ROC curves for prediction of postoperative pneumonia on one of the test data set. Greater AUC shows higher discriminative ability of the model. *AUC* area under the receiver operating characteristic curve, *SVM* support vector machine, *GBM* gradient boosting machine

Six machine learning models, including LR, SVM, RF, MLP XGBoost, and GBM, were constructed, and their performance for prediction of postoperative pneumonia was assessed. Additional file 1: Table S1 and Fig. 3 showed the best hyperparameter combination for each model and their AUCs in predicting postoperative pneumonia. XGBoost had the highest AUC value (0.793) with the lowest AUC value (0.674) for SVM. The AUC values of LR, SVM, and MLP were relatively lower than other three machine learning models. Ensemble machine learning models such as XGBoost, RF, and GBM showed significantly higher AUC values compared with LR, SVM, and MLP.

In addition to AUCs, accuracy, sensitivity, and specificity were used for evaluation of performance of the six machine learning models. As shown in Table 4, on the 1000 bootstraped test data sets, XGBoost model showed both best AUC (median, 0.794; 95% CI 0.735–0.84) and highest specificity (median, 0.815; 95% CI (0.75–0.872) among the machine learning models. The random forest model showed both best accuracy (median, 0.736; 95% CI 0.674–0.787) and highest sensitivity (median, 0.632; 95% CI (0.538–0.72) among the machine learning models (Table 4).

**Table 4:** Performance of the six ML models in the testing set

	AUC (95% CI)	Accuracy (95% CI)	Sensitivity (95% CI)	Specificity (95% CI)
LR	0.68 (0.607, 0.743)	0.657 (0.596, 0.713)	0.494 (0.405, 0.595)	0.778 (0.711, 0.84)
SVM	0.676 (0.606, 0.741)	0.646 (0.59, 0.702)	0.62 (0.529, 0.707)	0.67 (0.589, 0.746)
RF	0.781 (0.719, 0.833)	0.736 (0.674, 0.787)	0.632 (0.538, 0.72)	0.813 (0.747, 0.876)
MLP	0.678 (0.611, 0.744)	0.635 (0.579, 0.691)	0.514 (0.423, 0.603)	0.728 (0.651, 0.798)
GBM	0.772 (0.714, 0.827)	0.713 (0.657, 0.77)	0.605 (0.507, 0.697)	0.794 (0.723, 0.856)
XGBoost	0.794 (0.735, 0.84)	0.73 (0.674, 0.781)	0.618 (0.527, 0.705)	0.815 (0.75, 0.872)

Values are expressed as median with interquartile range

*LR* logistic regression, *SVM* support vector machine, *RF* random forest, *MLP* multilayer perceptron, *GBM* gradient boosting machine, *XGB* extreme gradient boosting

### Effect of postoperative pneumonia on patient outcomes

**Fig. 4:**
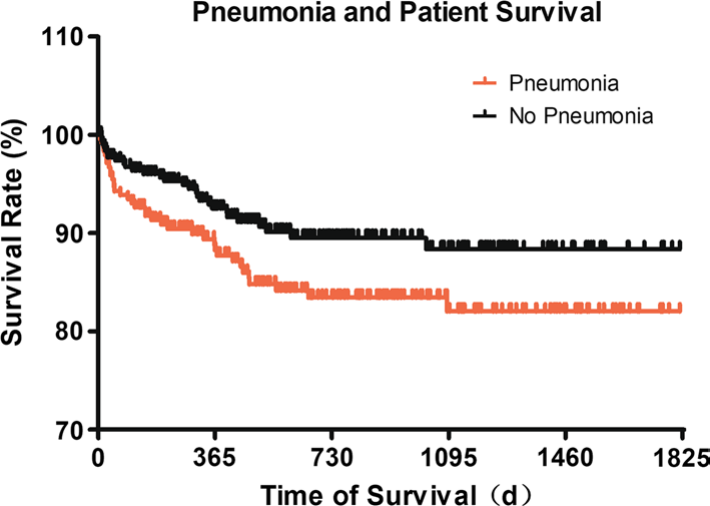
Survival rates of patients with or without postoperative pneumonia. 591 cases with a survival data that last over a 5-year interval were analyzed. The difference of both curves were examined by Log-rank test (Chi square 4.034, df 1, *P* = 0.0446) and Gehan–Breslow Wilcoxon test (Chi square 4.288, df 1, *P* = 0.0384)

Compare with the non-pneumonia group, the pneumonia group had longer postoperative hospital stay [22 (17, 31) *vs.* 23 (17, 33) days, *P* = 0.046] (Table 4) and lower 6-month, (91.0% vs. 96.2%; *P* = 0.01), 12-month (88.6% vs. 93.4%; *P* = 0.0045), 2-year (85.3% vs. 91.5%; *P* = 0.021), and 3-year (84.9% vs. 90.9%; *P* = 0.03) survival rates and overall survival rates (*P* = 0.0446; Table 5, Fig. 4) than patients without occurring postoperative pneumonia.

**Table 5:** Comparison of survival rate of the study patients

Survival duration	Patients without pneumonia (338)	Patients with pneumonia (253)	*P*_value
30 days	331 (97.93%)	245 (96.84%)	0.404
3 months	328 (97.04%)	237 (93.68%)	0.048
6 months	326 (96.45%)	231 (91.30%)	0.008
12 months	317 (93.79%)	225 (88.93%)	0.034
3 years	309 (91.42%)	216 (85.38%)	0.021

Data were expressed as frequency (proportion)

## Discussion

Early detection of postoperative pneumonia is critical for timely interventions to prevent the onset of the complication. Until now, the predication of postoperative pneumonia has been challenging, and there is need for reliable and accurate predictive model for patients after liver transplantation. This study, based upon large volume of data and ML methods, has the following major novel findings: (1) The incidence of postoperative pneumonia was high in patients after OLT, and the occurrence was significantly associated with prolonged hospital stay and increased mortality after liver transplantation; (2) A total of 14 factors were identified to be significantly correlated with postoperative pneumonia after OLT, including INR, HCT, PLT, ALB, ALT, FIB, WBC, PT, serum Na^+^, TBIL, anesthesia time, preoperative length of hospital stay, total fluid transfusion, and operation time; (3) The XGBoost model exhibited the best overall performance in predicting postoperative pneumonia among the developed ML models, with the value of AUC of 0.794, sensitivity of 52.6%, and specificity of 77.5%; (4) Multiple lines of evidence support that the XGBoost model holds promise for future clinical application in predicting postoperative pneumonia in patients after liver transplantation.

XGBoost model is recognized as an efficient and scalable tree boosting system [26], and it has performed well in the ML competitions, especially the simplicity in use and the accuracy in prediction [27, 28]. In the present study, we developed a total of six ML models, of these, XGBoost model had the best overall performance, with a specificity of 77.5% and a sensitivity of 52.6% in predicting postoperative pneumonia in OLT patients. In the study, the AUC values of LR, SVM, and MLP were relatively lower than other three ensemble machine learning models including XGboost, RF and GBM, whose accuracy and robustness might be attributed to their nature of integrating multiple base classifiers or learners. However, RF is a bagging ensemble, and it needs to train a large amount of decision trees and aggregate them. As a result, it usually takes much more time to trade numerous random computations for high accuracy, compared with GBM and XGboost, which both belong to boosting ensemble method. Moreover, compared to GBM, XGboost leverages second order derivative and implements sampling method in each iteration to alleviate overfitting and speed up computation.

Considering the high prevalence of multi-drug resistant bacteria in post-transplant patients induced by the excessive use of antibiotics [4], high specificity is especially necessary in clinical practice to avoid an unnecessary and overuse of antibiotics in low-risk patients. By contrast, all patients received peri-operative antibiotic therapy for 72 h, and this has posed considerable challenge in predicting pneumonia at an early stage [29]. Therefore, the novel XGBoost model as established in this study may assist clinicians in making optimal interventions and treatments, and eventually improve care for affected patients.

It has been reported that a number of risk factors, including age of recipient, liver dysfunction score, indication for OLT, perioperative transfusions especially the blood and fresh frozen plasma units, restrictive preoperative pulmonary testing pattern and INR measured prior OLT, are significantly associated with post-liver transplant pneumonia [3, 30, 31]. However, these factors are limited for its underutilization of within-category information, causing a loss of information [32]. For instance, patients above or below the optimal cut-point value had been equally considered in the risk-factor prediction, yet the risk of post-transplant pneumonia may vary considerably. As the risk-factor prediction is developed with neither combining all factors together nor weighting difference between different factors, it is not widely used in clinical practice. In addition, the traditional scores were given on the basis of the assumption that all misclassification errors have equal costs. In fact, this assumption is indefensible if apply in real-world applications [33]. In this study, we applied RFE feature selection method on 33 features which were statistically significant, of which 14 best features with the highest sensitivity score, including preoperative laboratory results of INR, HCT, PLT, ALB, ALT, FIB, WBC, PT, serum Na^+^, TBIL, anesthesia time; preoperative length of hospital stay, total fluid transfusion, and operation time. We found that most of the factors have been reported to be associated with pneumonia and PPCs except for PLT and serum Na^+^ [18, 30, 31, 34, 35]. As the risk factors reported in different literatures are quite different and this may be attributed to different population and definition of pneumonia and PPCs, we think it just reflects the advantage of ML models to capture previously unknown correlations in big data. Although the underlying mechanism remained unclear, the high clinical relevance of these factors laid a solid foundation for the consequent ML process and made the conclusion more practical and clinically valuable [36]. Moreover, we found the 14 features in ML model were all routinely recorded and widely used, and no factors need special instrument or equipment to obtain, indicating that our models are feasible and can be widely used in hospitals.

To date, ML models have shown outstanding performance in prediction of diseases and clinical conditions, for which these models can be helpful in decision-making about the use of interventions and medications [33]. For example, ML models can generate an individualized probability for each patient. Additionally, implementation of sophisticated computer algorithms at the bedside has become a reality since the popularity of EPR systems and wide availability of structured patient data. In our study, the EPR systems included HIS, LIS, PACS, and Docare Anesthesia System, which allowed us to integrate medical data generated during admission, covering demographic data, daily documentation, laboratory and imaging results, anesthesia records and thorough record of medication, and treatment. In addition, we separated the patients 1000 times (70% train and 30% test) into 1000 different pairs of train and test sets and this could minimize accidental error and enhance the accuracy of the current ML models. This result showed that in predicting post-transplant pneumonia, we should not apply only one of the ML model.

In the study, we found that patients with hepatic malignancy, better hepatic function before surgery, and longer hospital stay before surgery were significantly associated with lower risk of developing postoperative pneumonia. We postulated that this could be attributed to the better preoperative treatment and preparation, suggested that interventions should be implemented to improve the patients’ overall preoperative conditions. In consistence with previous reports [37, 38], we identified that a number of intraoperative factors, such as the longer operation and anesthesia time, excessive blood product transfusion, and fluid transfusion, were significantly related to postoperative pneumonia in patients following liver transplantation. By contrast, we found that there was an association between the use of telipressin and dopamine and decreased incidence of postoperative pneumonia in patients after liver transplantation. These findings are clinically important for the intraoperative anesthetic management and help improving the clinical outcomes.

The study may have several limitations. Firstly, the ML models are developed on the basis of a single-center cohort study, and future multi-center study will be needed for external validation. Secondly, this study is performed retrospectively, for which collection and entry bias, as well as possible residual confounding may occur. Thirdly, we were unable to incorporate the metrics of liver donors as training variables in our study, due to the lack of donor information in the EPR systems of our hospital.

## Summary

Our study has successfully established six novel ML models to predict postoperative pneumonia among OLT patients. Of these, the XGboost model has demonstrated overall best performance, and therefore holds promise for future clinical application to predict post-transplant pneumonia in OLT patients. To the best of our knowledge, this is the first ML-based study to provide a novel ML algorithm for prediction of postoperative pneumonia in patients after liver transplantation.

## Supplementary Information

**Additional file 1**: **Table S1. **Hyperparameter combinations for each machine learning models and their corresponding test set AUC.

## Data Availability

All data generated or analysed during this study are included in this published article [and its additional information files].

## Abbreviations

PPC: Postoperative pulmonary complications
OLT: Orthotopic liver transplantation
ML: Machine learning
LR: Logistic regression
SVM: Support vector machine
RF: Random forest
AdaBoost: Adaptive boosting
XGBoost: Extreme gradient boosting
GBM: Gradient boosting machine
ISAN: National Institutes of Health Stroke Scales
EPR: Electronic patient record
AKI: Acute kidney injury
LDLT: Living donor liver transplantation
DDLT: Deceased donor liver transplantation
HIS: Hospital information system
LIS: Laboratory information system
PACS: Picture archiving and communication system
TRIPOD: Transparent Reporting of a Multivariable Prediction Model for Individual Prognosis or Diagnosis
EPCO: European Perioperative Clinical Outcome
WBC: White blood cell
DCD: Donation of circulatory death
DBD: Donation of brain death
CBP: Continuous blood purification
ABP: Arterial blood pressure
CO: Cardiac output
SVV: Stroke volume variation
EBL: Estimated blood loss
TEG: Thromboelastogram
ICU: Intensive care unit
ALD: Alcohol-related liver disease
DILI: Drug-induced liver injury
RFE: Recursive feature elimination
MLP: Multilayer perceptron
GBM: Gradient boosting machine
CI: Confidence interval
AUC: Area under receiver-operating curve
SD: Standard deviation
HCT: Hematocrit
ALT: Alanine transaminase
TBIL: Total bilirubin
ALB: Albumin
RBC: Red blood cell
INR: International normalized ratio
PLT: Platelets
FIB: Fibrinogen
PT: Prothrombin time

## References

[1] Magalhães CBA, Nogueira IC, Marinho LS, Daher EF, Garcia JHP, Viana CFG, de Bruin PFC, Pereira EDB (2017). Exercise capacity impairment can predict postoperative pulmonary complications after liver transplantation. Respiration.

[2] Li X, Wei X, Chen C, Zhang Z, Liu D, Hei Z, Yao W. *N*-Acetylcysteine inhalation improves pulmonary function in patients received liver transplantation. Biosci Rep. 2018;38(5). 10.1042/BSR20180858 PMC6165840 30217943

[3] Levesque E, Hoti E, Azoulay D, Honore I, Guignard B, Vibert E, Ichai P, Antoun F, Saliba F, Samuel D (2012). Pulmonary complications after elective liver transplantation-incidence, risk factors, and outcome. Transplantation.

[4] Freire MP, Villela Soares Oshiro IC, Bonazzi PR, Pierrotti LC, de Oliveira LM, Machado AS, Van Der Heijdenn IM, Rossi F, Costa SF, Carneiro D’Albuquerque LA, Abdala E (2017). Surveillance culture for multidrug-resistant gram-negative bacteria: performance in liver transplant recipients. Am J Infect Control.

[5] Miguel Montanes R, Elkrief L, Hajage D, Houssel P, Fantin B, Francoz C, Dreyfuss D, Ricard JD, Durand F (2018). An outbreak of *Pneumocytis jirovecii* pneumonia among liver transplant recipients. Transpl Infect Dis.

[6] Miskovic A, Lumb AB (2017). Postoperative pulmonary complications. Br J Anaesth.

[7] Smith CJ, Bray BD, Hoffman A, Meisel A, Heuschmann PU, Wolfe CD, Tyrrell PJ, Rudd AG (2015). Can a novel clinical risk score improve pneumonia prediction in acute stroke care? A UK multicenter cohort study. J Am Heart Assoc.

[8] Arozullah AM, Khuri SF, Henderson WG, Daley J (2001). Development and validation of a multifactorial risk index for predicting postoperative pneumonia after major noncardiac surgery. Ann Intern Med.

[9] Shoka M, Kanda M, Ito S, Mochizuki Y, Teramoto H, Ishigure K, Murai T, Asada T, Ishiyama A, Matsushita H, Tanaka C, Kobayashi D, Fujiwara M, Murotani K, Kodera Y (2020). Systemic inflammation score as a predictor of pneumonia after radical resection of gastric cancer: analysis of a multi-institutional dataset. Dig Surg.

[10] Kostakis ID, Sotiropoulos GC, Kouraklis G (2014). *Pneumocystis jirovecii* pneumonia in liver transplant recipients: a systematic review. Transplant Proc.

[11] Nishi H, Oishi N, Ishii A, Ono I, Ogura T, Sunohara T, Chihara H, Fukumitsu R, Okawa M, Yamana N, Imamura H, Sadamasa N, Hatano T, Nakahara I, Sakai N, Miyamoto S (2019). Predicting clinical outcomes of large vessel occlusion before mechanical thrombectomy using machine learning. Stroke.

[12] Macesic N, Bear Don’t Walk OI, Peer I, Tatonetti NP, Peleg AY, Uhlemann AC (2020). Predicting phenotypic polymyxin resistance in *Klebsiella pneumoniae* through machine learning analysis of genomic data. mSystems.

[13] Quesada JA, Lopez-Pineda A, Gil-Guillen VF, Durazo-Arvizu R, Orozco-Beltran D, Lopez-Domenech A, Carratala-Munuera C (2019). Machine learning to predict cardiovascular risk. Int J Clin Pract.

[14] Artzi NS, Shilo S, Hadar E, Rossman H, Barbash-Hazan S, Ben-Haroush A, Balicer RD, Feldman B, Wiznitzer A, Segal E (2020). Prediction of gestational diabetes based on nationwide electronic health records. Nat Med.

[15] Li X, Wu M, Sun C, Zhao Z, Wang F, Zheng X, Ge W, Zhou J, Zou J (2020). Using machine learning to predict stroke-associated pneumonia in Chinese acute ischaemic stroke patients. Eur J Neurol.

[16] Luo Y, Tang Z, Hu X, Lu S, Miao B, Hong S, Bai H, Sun C, Qiu J, Liang H, Na N (2020). Machine learning for the prediction of severe pneumonia during posttransplant hospitalization in recipients of a deceased-donor kidney transplant. Ann Transl Med.

[17] Spann A, Yasodhara A, Kang J, Watt K, Wang B, Goldenberg A, Bhat M (2020). Applying machine learning in liver disease and transplantation: a comprehensive review. Hepatology.

[18] Li X, Chen C, Wei X, Zhu Q, Yao W, Yuan D, Luo G, Cai J, Hei Z (2018). Retrospective comparative study on postoperative pulmonary complications after orthotopic liver transplantation using the Melbourne Group Scale (MGS-2) diagnostic criteria. Ann Transplant.

[19] Darst BF, Malecki KC, Engelman CD (2018). Using recursive feature elimination in random forest to account for correlated variables in high dimensional data. BMC Genet.

[20] Yang HC, Islam MM, Nguyen PAA, Wang CH, Poly TN, Huang CW, Li YJ (2021). Development of a web-based system for exploring cancer risk with long-term use of drugs: logistic regression approach. JMIR Public Health Surveill.

[21] Wang RZ, Sun CH, Schroeder PH, Ameko MK, Moore CC, Barnes LE. Predictive models of sepsis in adult ICU patients. In: IEEE international conference on healthcare informatics (ICHI), New York, NY, USA. 2018:390–1

[22] Zhang K, Liu X, Jiang J, Li W, Wang S, Liu L, Zhou X, Wang L (2019). Prediction of postoperative complications of pediatric cataract patients using data mining. J Transl Med.

[23] Nasser IM, Abu-Naser SS (2019). Lung cancer detection using artificial neural network. Int J Eng Inf Syst.

[24] Zeng X, An J, Lin R, Dong C, Zheng A, Li J, Duan H, Shu Q, Li H (2020). Prediction of complications after paediatric cardiac surgery. Eur J Cardiothorac Surg.

[25] Zhu SL, Dong J, Zhang C, Huang YB, Pan W (2020). Application of machine learning in the diagnosis of gastric cancer based on noninvasive characteristics. PLoS ONE.

[26] Friedman JH (2001). Greedy function approximation: a gradient boosting machine. Ann Stat.

[27] Sheridan RP, Wang M, Liaw A, Ma J, Gifford E (2020). Correction to extreme gradient boosting as a method for quantitative structure-activity relationships. J Chem Inf Model.

[28] Adam-Bourdarios C, Cowan G, Germain-Renaud C, Guyon I, Kégl B and Rousseau D. The Higgs machine learning challenge. J Phys Conf Ser. 2015;664

[29] Li F, Wang C, Liu X, Peng Y, Jin S (2018). A composite model of wound segmentation based on traditional methods and deep neural networks. Comput Intell Neurosci.

[30] El-Badrawy MK, Ali RE, Yassen A, AbouElela MA, Elmorsey RA (2017). Early-onset pneumonia after liver transplant: microbial causes, risk factors, and outcomes, Mansoura University, Egypt, Experience. Exp Clin Transplant.

[31] Koo HJ, Lee HN, Choi SH, Sung H, Oh SY, Shin SY, Kim HJ, Do KH (2018). Human metapneumovirus infection: pneumonia risk factors in patients with solid organ transplantation and computed tomography findings. Transplantation.

[32] Chen CY, Lin WC, Yang HY (2020). Diagnosis of ventilator-associated pneumonia using electronic nose sensor array signals: solutions to improve the application of machine learning in respiratory research. Respir Res.

[33] Deo RC (2015). Machine learning in medicine. Circulation.

[34] Zhu M, Zhu Y, Guo F, Zhang J, Liu W, Hou W (2020). Clinical and laboratory characteristics of 215 cases of coronavirus disease 2019 with different prognosis. Zhonghua Wei Zhong Bing Ji Jiu Yi Xue.

[35] Li D, Liu C, Liu J, Hu J, Yang Y, Zhou Y (2020). Analysis of risk factors for 24 patients with COVID-19 developing from moderate to severe condition. Front Cell Infect Microbiol.

[36] Hu Z, Melton GB, Moeller ND, Arsoniadis EG, Wang Y, Kwaan MR, Jensen EH, Simon GJ (2016). Accelerating chart review using automated methods on electronic health record data for postoperative complications. AMIA Annu Symp Proc.

[37] Nijbroek SG, Schultz MJ, Hemmes SNT (2019). Prediction of postoperative pulmonary complications. Curr Opin Anaesthesiol.

[38] Siniscalchi A, Aurini L, Benini B, Gamberini L, Nava S, Viale P, Faenza S (2016). Ventilator associated pneumonia following liver transplantation: etiology, risk factors and outcome. World J Transplant.

