# Supplementary material for: Development and performance assessment of novel machine learning models to predict pneumonia after liver transplantation
Source: Respir Res. 2021 Mar 31;22:94. doi: 10.1186/s12931-021-01690-3 (PMC8011203; doi:10.1186/s12931-021-01690-3)
Supplement: Supplementary file 1 — Additional file 1: Table S1. Hyperparameter combinations for each machine learning models and their corresponding test set AUC. [file 12931_2021_1690_MOESM1_ESM.doc]

**Additional information**

**Table S1. Hyperparamter combinations for each machine learning models and their corresponding test set AUC**

|  | **Hyperparameters Set** | **Optimal Hyperparameters** | AUC |
| --- | --- | --- | --- |
| Models |  |  | 0.455 |
| LR | L1 Regularization term: [0.0001, 0.0008, 0.006, 0.046,0.36, 2.78, 21.54, 168.81,1291.54, 10000]; Smaller value specifies greater regularization strength | L1 Regularization term: 21.54 | 0.680 |
| SVM | Kernel:{Linear, Polynomial, Radial Basis, Sigmoid} Regularization term: {0,1,2,3,4,5} | Kernel: Radial Basis Regularization term: 2 | 0.674 |
| RF | Number of estimators:{50,100,500} Maximum depth:{None,2,4,6,8} | Number of estimators: 100 Maximum depth: None | 0.781 |
| GBM | Number of estimators: {50,100,500} Learning rate: {0.1,0.05,0.01} | Number of estimators: 500 Learning rate: 0.05 | 0.771 |
| MLP | Batch size:{16,64,128} Learning rate method:{Constant, Decay} Initial learning rate:{0.01,0.001,0.0001} L2 Regularization term:{0.001,0.01,0.1,1}; Larger alpha specifies larger regularization | Hidden layer size:{(7,2)} Optimization method: Adam  Activation function: ReLU Batch size: 16 Learning rate method: Decay Initial Learning rate: 0.01 L2 Regularization term: 0.1 | 0.676 |
| XGB | Number of estimators: {50,100,200,500} Learning rate: {0.1,0.05,0.01} Maximum depth: {3,4,5,6} | Number of estimators: 500 Learning rate: 0.01 Maximum depth: 3 | 0.793 |

Note: LR, logistic regression; SVM, support vector machine; RF, random forest; MLP, multilayer perceptron; GBM, gradient boosting machine; XGB, extreme gradient boosting. AUC score measured the model performance on testing set.
